# Transcription Factor *Mc*HB7 Improves Ice Plant Drought Tolerance through ABA Signaling Pathway

**DOI:** 10.3390/ijms25084569

**Published:** 2024-04-22

**Authors:** Xuemei Zhang, Zihan Cheng, Gaofeng Fan, Dan Zhu, Bowen Tan, Tingbo Jiang, Sixue Chen

**Affiliations:** 1 College of Horticulture and Gardening, Yangtze University, Jingzhou 434025, China; echozhang@yangtzeu.edu.cn (X.Z.); 522046@yangtzeu.edu.cn (Z.C.); 2 State Key Laboratory of Tree Genetics and Breeding, Northeast Forestry University, Harbin 150040, China; fgf15935406410@sina.com (G.F.); tbjiang@nefu.edu.cn (T.J.); 3 Department of Biology, Genetics Institute, University of Florida, Gainesville, FL 32610, USA; zhudandora@qau.edu.cn (D.Z.); btan@go.olemiss.edu (B.T.); 4 College of Life Sciences, Qingdao Agricultural University, Qingdao 266109, China; 5 Department of Biology, University of Mississippi, Oxford, MS 38677, USA

**Keywords:** *Mesembryanthemum crystallinum*, *McHB7*, drought stress, proteomics, metabolomics, ABA signaling

## Abstract

As global climate change continues, drought episodes have become increasingly frequent. Studying plant stress tolerance is urgently needed to ensure food security. The common ice plant is one of the model halophyte plants for plant stress biology research. This study aimed to investigate the functions of a newly discovered transcription factor, Homeobox 7 (HB7), from the ice plant in response to drought stress. An efficient *Agrobacterium*-mediated transformation method was established in the ice plant, where ectopic *McHB7* expression may be sustained for four weeks. The *McHB7* overexpression (OE) plants displayed drought tolerance, and the activities of redox enzymes and chlorophyll content in the OE plants were higher than the wild type. Quantitative proteomics revealed 1910 and 495 proteins significantly changed in the OE leaves compared to the wild type under the control and drought conditions, respectively. Most increased proteins were involved in the tricarboxylic acid cycle, photosynthesis, glycolysis, pyruvate metabolism, and oxidative phosphorylation pathways. Some were found to participate in abscisic acid signaling or response. Furthermore, the abscisic acid levels increased in the OE compared with the wild type. *Mc*HB7 was revealed to bind to the promoter motifs of Early Responsive to Dehydration genes and abscisic acid-responsive genes, and protein–protein interaction analysis revealed candidate proteins responsive to stresses and hormones (e.g., abscisic acid). To conclude, *McHB7* may contribute to enhance plant drought tolerance through abscisic acid signaling.

## 1. Introduction

*Mesembryanthemum crystallinum* (common ice plant) is native to the Namib Desert in South Africa [1]. Compared with other crassulacean acid metabolism (CAM) plants, it grows relatively fast and produces a lot of seeds. As a model facultative plant that can shift from C_3_ photosynthesis to CAM, ice plants are conducive to grow in low-water-potential soil [2]. Due to its ability to change the mode of photosynthesis during stresses (e.g., salt stress and drought), the ice plant has been widely used to study plant stress response mechanisms. In recent years, drought stress has affected more than 20% of arable land, leading to more than a 50% decrease in crop yield [3,4]. It is therefore vital to uncover the mechanisms of plant drought response and enhance plant stress resilience in the ever-changing environment.

Transcription factors (TFs) are regulatory proteins that interact with *cis*-elements of target genes to regulate their expression in many biological processes (e.g., abiotic stress responses) [4,5]. In a previous transcriptomic study, several ice plant TFs were found to be differentially expressed during the C_3_ to CAM transition induced by salt stress including bHLH (comp14048_c0_seq1, Mcr010456.002, and Mcr014957.003), NAC (comp21521_c0_seq1 and Mcr002150.001), GRAS (Contig16446), WRKY (Contig20720), and Homeobox TF (Contig9771) [6]. During the transition, a Homeobox 7 gene (*McHB7*) was significantly increased [6]. *McHB7* belongs to the Homeobox TF family, which is characterized by its helix-loop-helix-turn-helix structure homeodomain (HD) across different genera including plants, insects, animals, and humans [7]. Many homeobox genes have been reported to be involved in plant growth and development as well as stress response. For example, the rice *WUSCHEL*-related homeobox genes participate in plant reproductive organ development, hormone signaling, and abiotic stress response [8]. In pinesap (*Monotropa hypopitys*), *MhyWOX13* plays a role in the root stem cell niche, seed pod formation, flower initiation, and basic cellular processes [9]. In apple, *MdHOXs* have been shown to be involved in abiotic stress response, as *MdHOXs* were upregulated in the leaves and roots after treatments with cold, salt or abscisic acid (ABA) [10]. In Arabidopsis, *AtHB17* was found to coordinate the expression of photosynthesis-related nuclear genes and sigma factor *AtSIG5* to enhance stress tolerance [11]. Recent studies have confirmed that the overexpression of *McHB7* in ice plants and Arabidopsis showed improved salt tolerance [12,13], but the functions of *McHB7* in response to drought and the mechanism of drought tolerance in ice plants are unknown.

*Agrobacterium*-mediated T-DNA transfer from *Agrobacterium* to plant cells has been commonly used in producing a variety of transgenic plants [14] (e.g., Arabidopsis [15], tobacco [16], poplar [17], and rapeseed [18]). Recently, Agarie [19] and Hwang [20] used *Agrobacterium*-mediated transformation to obtain transgenic callus and roots of ice plants. In this study, *McHB7* was overexpressed in the ice plant leaves. To reveal molecular changes following the overexpression (OE) of *McHB7*, the transgenic leaf materials were used for global proteomics and metabolomics, which have shown utility in understanding how plants respond to abiotic stresses [21,22]. Many proteins or metabolites in different pathways have been found to be differentially changed in the *McHB7* OE plants under control and drought stress. Among them, ABA was increased in the *McHB7* OE plants compared to the wild type (WT). With yeast one-hybrid analysis and protein–protein interaction analysis, multiple lines of evidence have been provided to support the hypothesis that *McHB7* contributes to ice plant drought tolerance through ABA signaling, which may be activated by *Mc*HB7.

## 2. Results

### 2.1. Transformation of Ice Plant Leaves with GFP and McHB7

**Figure 1:**
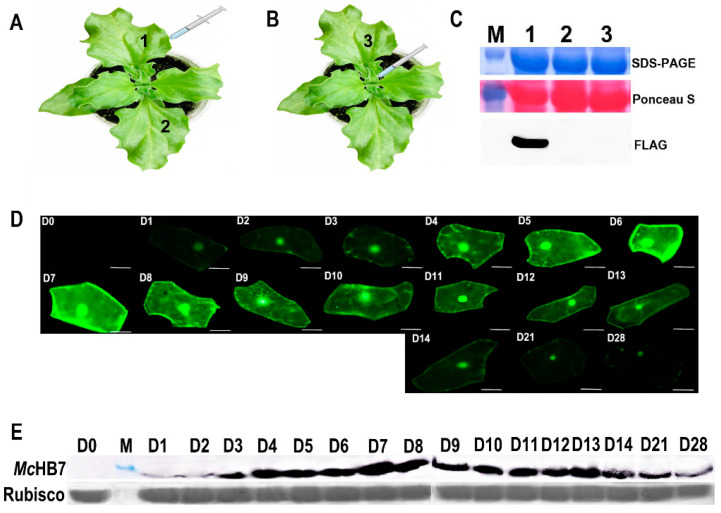
Identification of *McHB7*-overexpressing (OE) transgenic ice plant leaves. (**A**) Infiltration for ice plant transformation (1—one of the second pair of leaves from one-month-old ice plants was used for *Agrobacterium* infiltration and the infiltrated leaf was collected for protein extraction; 2—the non-infiltrated leaf was collected for protein extraction). (**B**) The stem was infiltrated and the second pair of leaves—3 was collected for protein extraction. (**C**) Western blot analysis of extracted proteins from the ice plant leaves 1–3. (**D**) Temporal changes of the GFP signal in the epidermal cells after infiltration for up to 28 days. (**E**) Western blot of transgenic ice plant leaves from day 0 (D0) to day 28 (D28).

Ice plant leaves and stems were infiltrated with agrobacteria containing *pCAMBIA1300-McHB7-FLAG* (Figure 1A,B). The Western blot results showed the presence of the *Mc*HB7-FLAG protein only in the infiltrated leaves (Figure 1C). Additionally, the GFP signal in the epidermal cells was observed under a fluorescence microscope. As shown in Figure 1D, the GFP signal was observed on day 1 and gradually increased and reached the highest level on day 7, before the signal started to decrease. Nevertheless, the GFP signal in the leaves could be observed for up to four weeks. The long interval of expression allowed us to test the functions of *McHB7* in its native ice plant system.

Ice plant leaf samples transformed with a *pCAMBIA1300-McHB7-FLAG* construct were collected after 0 to 28 days for protein and RNA extraction. The Western blot showed that *Mc*HB7-FLAG was expressed in the ice plant for four weeks, and the protein had the highest level on days 7 or 8 after infiltration (Figure 1E). The RT-qPCR results suggest that the infiltration method allowed for *McHB7* overexpression for a long period of time, and that the relative expression level reached its peak on day 7 (Supplementary Figure S1). The long-term overexpression of *McHB7* in ice plants allows for the studying of stress responses, and the leaves on day 7 post infiltration were used for the omics experiments.

### 2.2. McHB7 OE Ice Plants under Drought Stress

**Figure 2:**
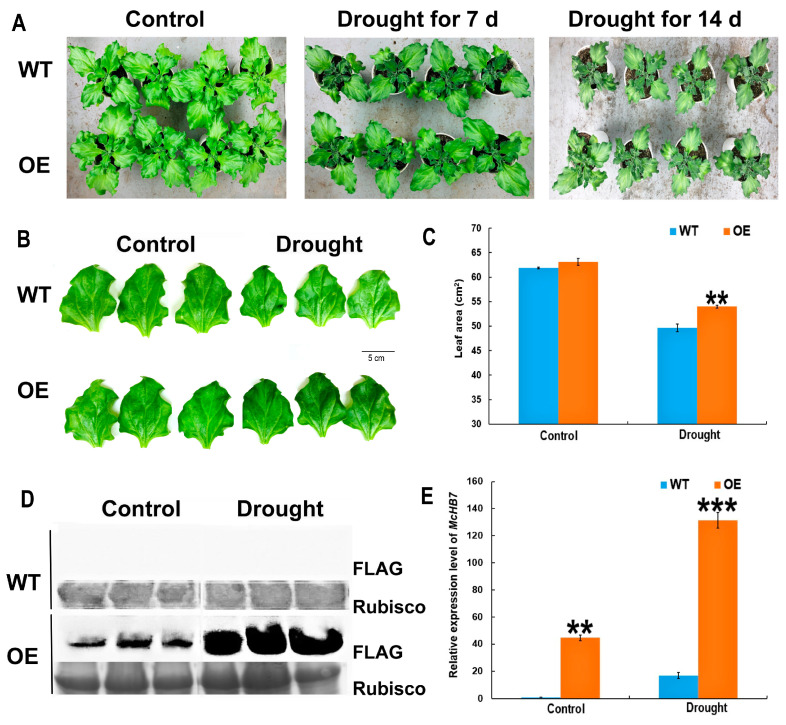
Characteristics of the *McHB7*-OE plants under control and drought conditions. (**A**) Phenotype of the OE and WT under control and drought stress. (**B**) Leaf phenotypic changes in the OE and WT after drought stress for 7 days. Bar = 5 cm. (**C**) Leaf areas of OE and WT under control and drought stress for 7 days. (**D**) *Mc*HB7 protein levels in the OE under the control and drought conditions for 7 days compared with the WT. Please see Supplementary Figure S2 for the whole gel images. (**E**) Relative expression levels of the *McHB7* in the OE under control and drought stress for 7 days compared with the WT. Student’s *t*-test: ** *p* < 0.01, *** *p* < 0.001. Error bars indicate mean ± SD.

To compare the differences between OE and WT under drought stress, the seedlings at day 7 post infiltration were treated with drought for one week and two weeks (Figure 2A). Under the control conditions, the growth of OE and WT plants was similar (Figure 2A). Under drought stress for 7 days, the OE plants showed higher chlorophyll levels than the WT (Figure 2B). The leaf areas of the OE plants were increased approximately 1.2 times compared to the WT (Figure 2C). Western blot results showed that *McHB7* had a higher expression level after drought stress than the control (Figure 2D). RT-qPCR results showed that the relative expression level of *McHB7* was 44.6 times in the OE lines compared with the WT under control conditions. Under drought, the relative expression level of *McHB7* was induced in the WT while in the OE plants, it was significantly elevated and reached 131.5 times that of the WT (Figure 2E).

### 2.3. Physiological Indices of McHB7 OE Plants under Drought Stress

**Figure 3:**
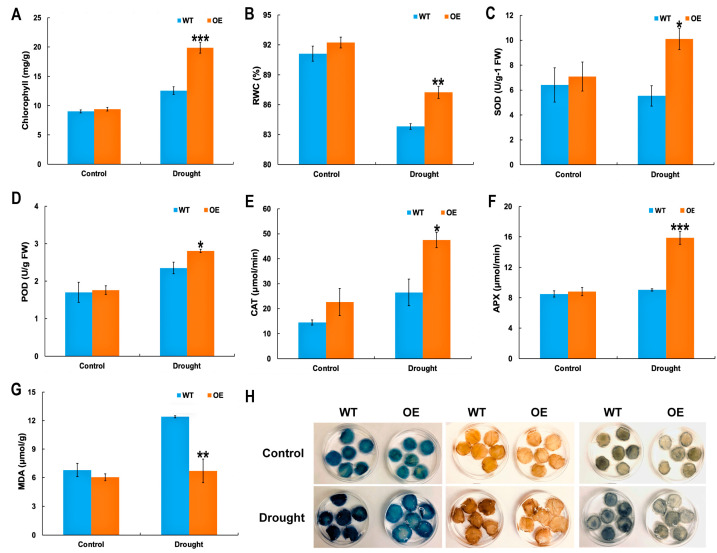
Physiological parameters of the *McHB7*-OE and WT under salt and drought stress. (**A**) Chlorophyll content. (**B**) Relative water content (RWC). (**C**) Superoxide dismutase (SOD). (**D**) Peroxidase (POD). (**E**) Catalase (CAT). (**F**) Ascorbate peroxidase (APX). (**G**) Malondialdehyde (MDA). (**H**) Histochemical staining of the OE and WT under control and drought stress. Student’s *t*-test: * *p* < 0.05; ** *p* < 0.01; *** *p* < 0.001. Error bars indicate mean ± SD.

Under the control conditions, the physiological parameters (e.g., chlorophyll content and RWC, activities of SOD, POD, CAT, and APX as well as contents of MDA) did not show significant differences between the OE and WT. However, under drought stress, the chlorophyll content in the OE was 1.6 times that of the WT (Figure 3A), the RWC was elevated by 4% in the OE compared with the WT (Figure 3B), and the SOD, POD, CAT, and APX activities were 1.8, 1.2, 1.8, and 1.8 times those of the WT, respectively (Figure 3C–F), while the MDA of WT was about twice that of the OE plants under drought stress (Figure 3G). Histochemical staining experiments with Evans blue, NBT, and DAB staining were conducted to detect cell death, hydrogen peroxide, and superoxide, respectively. As shown in Figure 3H, the leaves of the WT showed a darker blue color than OE under drought stress. Similar trends were observed for the DAB and NBT staining. The overall staining intensities of Evans blue, NBT, and DAB in the WT were significantly higher than those of the OE plants (Supplementary Figure S3). All the results suggest that the *McHB7* OE enhanced ice plant tolerance to drought stress.

### 2.4. Protein Changes in WT and OE Plants after Drought Stress

**Figure 4:**
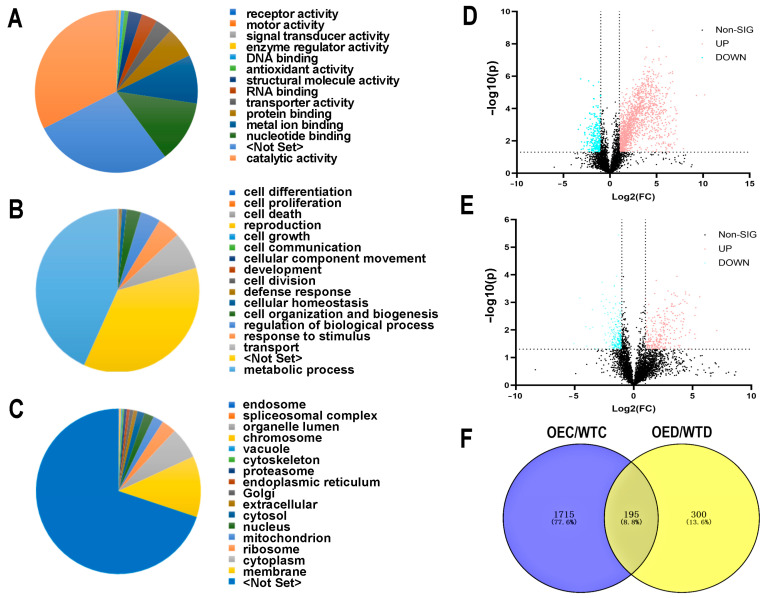
Protein changes in the *McHB7*-OE and WT under the control and drought conditions. (**A**) GO (Gene Ontology) annotation of molecular function. (**B**) GO annotation of biological process. (**C**) GO annotation of cellular component. (**D**) Differentially expressed proteins between the OE and WT under control conditions. (**E**) Differentially expressed proteins between the OE and WT under drought stress. (**F**) Venn diagram of differentially expressed proteins.

A total of 7053 proteins were identified in the ice plant leaves under the control and drought conditions. These proteins were involved in different molecular functions (Figure 4A) including catalytic activity (32.48%), nucleotide binding (12.43%), metal ion binding (9.70%), protein binding (6.12%), transporter activity (3.31%), RNA binding (3.31%), structural molecule activity (2.69%), antioxidant activity (0.82%), DNA binding (0.69%), enzyme regulator activity (0.38%), signal transducer activity (0.30%), motor activity (0.14%), and receptor activity (0.09%). For the biological process, the proteins were involved in the metabolic process (43.29%), transport (7.46%), response to stimulus (4.45%), regulation of biological processes (4.00%), cell organization and biogenesis (2.83%), cellular homeostasis (0.95%), defense response (0.34%), development (0.10%), and reproduction (0.04%) (Figure 4B). For the cellular component, the proteins were in the membrane (11.97%), cytoplasm (6.19%), ribosome (2.87%), mitochondrion (2.02%), nucleus (1.87%), cytosol (1.44%), extracellular (0.77%), Golgi (0.72%), endoplasmic reticulum (0.57%), proteasome (0.56%), cytoskeleton (0.33%), vacuole (0.27%), chromosome (0.21%), organelle lumen (0.11%), spliceosome complex (0.09%), and endosome (0.08%) (Figure 4C).

**Figure 5:**
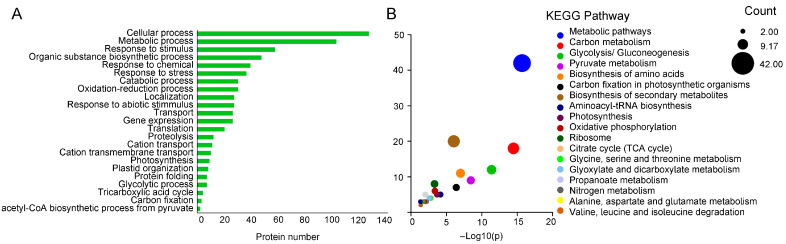
Differentially expressed proteins analysis. (**A**) GO predication of DEP (differentially expressed proteins). (**B**) KEGG pathway prediction of significantly increased proteins. Please note that academic users may freely use the KEGG website (https://www.kegg.jp, accessed on 30 November 2023).

For the identified proteins, 1910 were significantly different between the WT and OE under the control conditions (Fold change (FC) > 2, *p*-value < 0.05), among which 1617 were increased and 293 were decreased (Figure 4D). A total of 495 proteins were significantly changed in levels between the OE and WT under drought stress (FC > 2, *p*-value < 0.05), among which 255 were increased and 240 were decreased (Figure 4E). Based on the Venn diagram, there were 195 proteins differentially expressed and 137 were significantly increased in the OE compared with the WT under the control and drought conditions including pyruvate kinase (A0A087GXE3), malic enzyme (A0A178VRX4), glutathione synthetase (A0A1J3CTP6), calcium sensing receptor (A0A2G3CYX6), and phosphoenolpyruvate carboxylase (Q8H928) (Figure 4F). These functioned in different cellular or metabolic pathways including the pyruvate metabolic process (GO:0006090), TCA cycle (GO:0006099), oxidation–reduction process (GO:0055114), and response to abiotic stimulus (GO:0009628) (Figure 5A). Additionally, they were involved in 28 KEGG pathways, including pyruvate metabolism (ath00620), photosynthesis (ath00195), glycolysis/gluconeogenesis (ath00010), and oxidative phosphorylation (ath00190) (Figure 5B). Furthermore, 118 proteins were uniquely increased in the OE after drought stress, among which some were related to ABA (e.g., a lipid phosphate phosphatase 2 (LPP2) (A0A200QUV2) and a protein phosphatase 2C (A0A2I0WZU3)), indicating that *McHB7* might play a positive role in drought response through ABA signaling.

### 2.5. McHB7 Protein Binds to Dehydration and ABA-Related Cis-Acting Elements and Has Self-Transactivation

**Figure 6:**
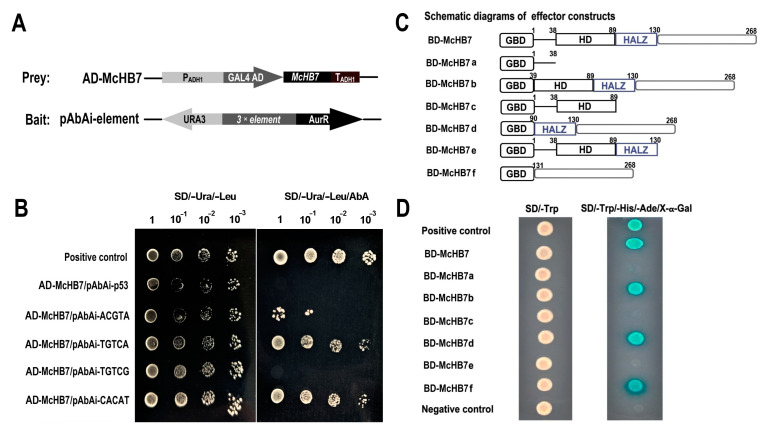
Characterization of *Mc*HB7 binding to dehydration and ABA-related *cis*-acting elements and self-transactivation. (**A**) Schematic illustration of the vector construction for the yeast one-hybrid analysis. (**B**) Yeast one-hybrid analysis of *Mc*HB7 binding to different *cis*-acting elements, ACGTA, TGTCG, TGTCA, and CACAT elements. (**C**) Schematics of vector construction for transactivation identification through a series of deletion of *Mc*HB7 domains. (**D**) Yeast two-hybrid analysis of the *Mc*HB7 self-transactivation domain from 131 to 268 aa.

Based on the proteomics data, it can be hypothesized that *Mc*HB7 may function through ABA signaling. This hypothesis is supported by the fact that the *Mc*HB7 homolog in Arabidopsis *At*HB7 (AT2G46680.1) binds to various *cis*-acting elements like ABA responsive ACGTA and TGTCG motifs, ERD motif TGTCA, and the ABA-related motif CACAT [23]. Thus, the yeast one-hybrid experiment was conducted to test the binding of *Mc*HB7 to the *cis*-acting elements (Figure 6A). As shown in Figure 6B, all of the transformant yeast cells grew well on the selective medium of SD/-Ura/-Leu. However, on the plate with 250 ng/mL Aureobasidin A (AbA), the positive controls, AD-*Mc*HB7/pAbAi-TGTCA and AD-*Mc*HB7/pAbAi-CACAT could grow normally, even at a dilution of 1000 times, but AD-*Mc*HB7/pAbAi-p53, AD-*Mc*HB7/pAbAi-TGTCG could not grow on the medium, and AD-*Mc*HB7/pAbAi-ACGTA could not grow when it was diluted 100 times. Clearly, *Mc*HB7 can specifically bind to the ERD motif TGTCA and the ABA-related motif CACAT, and its binding to another ABA *cis*-element, ACGTA, is relatively weak (Figure 6B). The results show that *Mc*HB7 may be involved in the ABA signaling pathway by binding to ABA-related *cis*-acting elements.

Through the transactivation assay, *Mc*HB7, as a TF, showed self-transactivation. A series of deletion analysis revealed that only a non-conserved domain from 131 to 268 amino acid (aa) in the C-terminus acted as a potent activator (Figure 6C,D). The analysis of the transactivation of *Mc*HB7 will help to further explore its interacting proteins.

### 2.6. Immunoprecipitation and Protein-Protein Interaction

**Figure 7:**
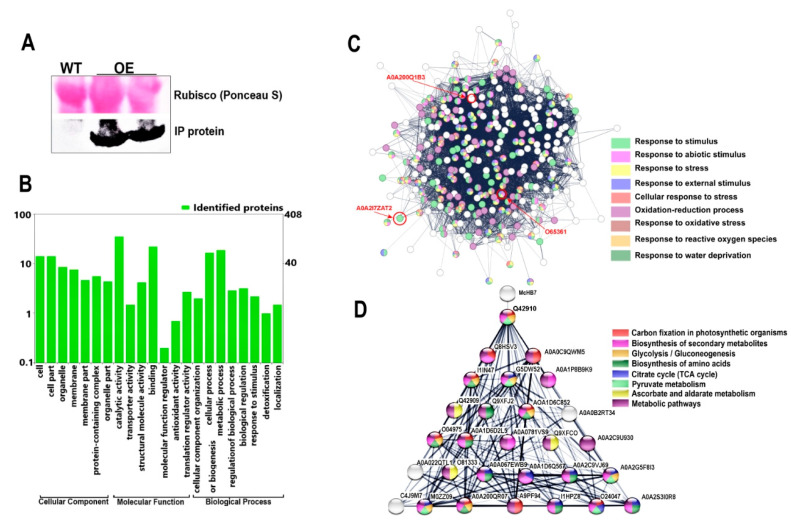
Protein–protein interaction (PPI) analysis of the identified proteins in the immunoprecipitation (IP) of the *Mc*HB7–FLAG complex. (**A**) IP result with the *Mc*HB7 bait. Top panel, input of WT and OE showing equal loading with the Rubisco large subunit; bottom panel, Western signal with the anti-FLAG antibody. (**B**) GO annotation of the 408 proteins in the *Mc*HB7 complex. (**C**) Predicted networks with the identified proteins by STRING; the red circles indicate the proteins related to ABA. (**D**) PPI network showing *Mc*HB7 interaction with Q42910 and its predicted protein interaction network.

To explore proteins potentially forming complexes with *Mc*HB7 in ice plants, immunoprecipitation (IP) with the *Mc*HB7-FLAG as bait was performed. As shown in Figure 7A, only the OE leaves had the corresponding band when the FLAG antibody was used in Western blot. The IP proteins were analyzed by LC-MS/MS, and 408 proteins were identified in the OE samples. GO enrichment analysis revealed that the proteins functioned in different biological processes including the response to stimulus, regulation of biological process, metabolic, and cellular processes (Figure 7B). Some proteins were involved in molecular function like antioxidant activity (which helps to maintain ROS homeostasis) and translation regulator activity. Some proteins were part of a cellular component such as a catalytic complex, chaperone complex, TCA enzyme complex, and ribonucleoprotein complex. According to the STRING analysis, most of these proteins may interact with each other and are involved in various pathways (Figure 7C) such as the response to water deprivation (GO:0009414), oxidation–reduction process (GO:0055114), response to oxidative stress (GO:0006979), response to stress (GO:0006950), and stimulus (GO:0009605). Among these proteins, some were found to be relative to ABA (Figure 7C) such as an inositol monophosphatase (A0A2I7ZAT2) that is involved in the response to ABA and dehydration [24], a P-loop containing nucleoside triphosphate hydrolase (A0A200Q1B3) as a positive regulator of ABA signaling [25], and a delta-1-pyrroline-5-carboxylate synthetase (O65361) induced by dehydration and ABA [26]. Additionally, *Mc*HB7 can directly interact with the nucleus-located protein Q42910, which may act as a pyruvate/phosphate dikinase and functions in the formation of phosphoenolpyruvate (PEP) (https://www.uniprot.org/uniprot/Q42910, accessed on 30 November 2023) (Figure 7D).

### 2.7. Metabolomic Changes after Treatment with Drought Stress

**Figure 8:**
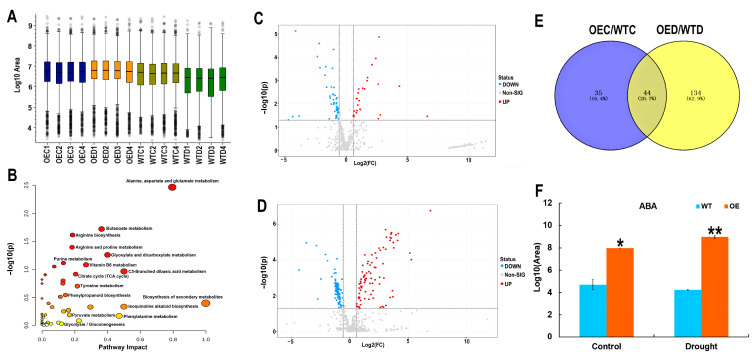
Metabolomic analysis of the OE and WT under the control and drought conditions. (**A**) Boxplot of the OE and WT samples under the control and drought conditions. (**B**) Pathway of identified metabolites. (**C**) Significantly changed metabolites in the OE and WT under control conditions. (**D**) Significantly changed metabolites in OE and WT under drought conditions. (**E**) Venn diagram of differentially changed metabolites under the control and drought conditions. (**F**) Changes in ABA in the OE and WT under the control and drought conditions. Student’s *t*-test: *, *p* < 0.05; **, *p* < 0.01. Error bars indicate mean ± SD.

Plant metabolites play an important role in stress response and adaptation, and specialized metabolites accumulate when plants are stressed [27]. Here, leaves from four replicates under the control and drought conditions were collected for metabolomic analysis (Figure 8A). A total of 514 metabolites were identified, which were involved in butanoate metabolism, arginine biosynthesis, the TCA cycle, phenylpropanoid biosynthesis, pyruvate metabolism, and biosynthesis of specialized metabolites (Figure 8B). A total of 79 and 178 metabolites were significantly changed between the OE and WT leaves under the control and drought conditions, respectively (Figure 8C,D) (*p* < 0.05). A total of 44 were identified to be differentially changed the OE compared with WT the (Figure 8E). These included maltose, lactose, jasmone, spermine, and cGMP. These metabolites are involved in riboflavin metabolism, histidine metabolism, starch and sucrose metabolism, beta-alanine metabolism, galactose metabolism, glutathione metabolism, purine metabolism, arginine and proline metabolism.

The ABA level was about 1.6 times higher in the OE than in the WT under the control conditions. Drought stress caused an additional increase in ABA in the OE leaves, almost twice as that of the WT (Figure 8F). This increase may contribute to the stress tolerance phenotypes and physiology of the OE plants (Figure 2 and Figure 3).

## 3. Discussion

The common ice plant is a stress-tolerant species that displays a well-characterized shift from C_3_ to CAM following high salinity, drought, and high light intensity [28,29]. Because of its ability to grow in high salinity and dry soil, the ice plant has been a model used to investigate the mechanisms of stress response and tolerance [30,31]. Based on our previous study [6], the transition from C_3_ to CAM in ice plants occurred after 500 mM NaCl treatment for seven days, and 18 TF genes were significantly changed during the transition. One of these TFs is *McHB7*, which has been shown to play a role in response to salt stress in ice plants [13] and Arabidopsis [12]. To investigate the molecular mechanisms underlying the *McHB7* function in response to drought stress, *McHB7* was successfully expressed in ice plant leaves for four weeks. This in planta long-term overexpression system not only allows for functional studies of *McHB7* in the same plant, but also avoids generating the tedious stable transformants through tissue culture. The method is novel in ice plants because in other species, the target gene expression often lasts for only a few days [32,33]. The overexpression of *McHB7* in ice plants did not show any obvious growth differences compared with the WT under normal conditions, but clearly contributed to the enhanced performance under the drought treatment condition. The OE leaves had increased chlorophyll content, RWC, SOD, POD, CAT, APX, and decreased MDA under drought stress (Figure 3).

The first homeobox gene was identified in *Drosophila* in 1983 [34], and then in plants in 1991 [35]. To date, many homeobox TFs have been discovered in different species. In plants, these have been divided into 14 classes including HD-ZIP I, HD-ZIP II, HD-ZIP III, HD- ZIP IV, KNOX, BEL, PLINC, WOX, DDT, PHD, NDX, LD, PINTOX, and SAWADEE [36]. However, there are no studies on ice plant homeobox TF genes. Here, *McHB7* was assigned to the HD-ZIP Ⅰ class based on its homology with Arabidopsis *AtHB7*. The yeast one-hybrid result showed that *Mc*HB7 can bind to the *cis*-elements TGTCA (with ERD motif) and CACAT (with ABA-related motif), suggesting that *Mc*HB7 may act to regulate the plant ABA-sensitivity in response to drought stress. According to the data of IP-MS, 408 proteins were identified in the OE leaves. Some of them were involved in removing ROS accumulated during the stress treatment. Thus, *Mc*HB7 may interact with these proteins to alleviate the damage from oxidative stress. Interestingly, some proteins were found to be related to ABA signaling or ABA biosynthesis. Furthermore, the metabolomics results showed increased ABA levels in the OE leaves and in the drought-treated plants. Together, these lines of evidence support an important function of *Mc*HB7 in ABA signaling, which is consistent with the function of *At*HB7 [23].

Oxidative stress occurs when ROS overwhelm the cellular antioxidant capacity to cause functional and structural damages [37]. It has been reported that SOD, POD, CAT, and APX are pivotal enzymes that act to scavenge ROS [38,39]. For example, SOD can convert superoxide radical to O_2_, APX can catalyze the conversion of H_2_O_2_ to H_2_O and O_2_ [40], and CAT also decomposes H_2_O_2_ to H_2_O and O_2_ [38]. POD can catalyze the process of H_2_O_2_ reduction by phenolic compounds, specialized metabolites, or other substrates [41]. MDA is cytotoxic and is associated with premature senescence, which can cause the cross-linking of macromolecules such as proteins and nucleic acids [42]. In the OE lines, SOD, POD, CAT, and APX activities were significantly elevated compared with the WT under drought stress, while the MDA content was lower in the OE than WT. Additionally, the histochemical staining images showed lower damage in the OE leaves than in the WT after drought stress. A possible explanation for these results is that an enhanced enzymatic antioxidant system may exist in the OE leaves to maintain redox homeostasis. Moreover, ROS may be part of the transition from C_3_ to CAM, as CAM acts as an important component in reducing the oxidative damage caused by stress [28,43]. In particular, H_2_O_2_ is reported to be a signaling molecule participating in cellular responses like promoting stress-related protein production and increasing antioxidant molecules [44]. The results of the lower ROS in the OE compared with the WT indicate that *McHB7* may contribute to protecting ice plants from ROS damage under stress conditions. Whether *McHB7* functions directly in the redox pathways or through ABA signaling deserves further investigation.

Proteomics and metabolomics have been useful in understanding the complex processes of plant biology [45,46]. In this study, IP-MS proteomics was utilized to discover the *Mc*HB7 complex proteins. For example, *Mc*HB7 can directly interact with nucleus-located protein Q42910, which participates in the synthesis of PEP, while PEP is related to carbon fixation, the TCA cycle, pyruvate metabolism, glycolysis/gluconeogenesis, ascorbate and aldarate metabolism, and the biosynthesis of secondary metabolites. PEP also plays a crucial role in the CAM pathway, suggesting that *Mc*HB7 may contribute to the transition of ice plants from C_3_ to CAM through interacting with Q42910. Interestingly, some identified proteins had antioxidant activity and are closely related to ABA, suggesting that *Mc*HB7, as a stress-induced TF, might interact with these proteins to promote or accelerate ROS reduction through the ABA signaling pathway. Additionally, after treatment with drought for seven days, the data showed that the differentially expressed proteins could participate in many molecular, cellular, and biological processes; in particular, many proteins are involved in the response to abiotic stimulus or stresses. These data support the function of *Mc*HB7 in modulating the expression of stress-related proteins and then enhancing the resistance to stress. Interestingly, some proteins were found to positively regulate the ABA-activated signaling pathway, for example, A0A200QUV2, with 78.4% similarity with the Arabidopsis homolog LPP2, is involved in ABA signaling, and A0A2I0WZU3, with 54.4% similarity with the Arabidopsis homolog PP2C5 phosphatase, also acts as a positive regulator in ABA-inducible gene expression. However, the causal effect relationship between these proteins and ABA still needs further investigation. The yeast one-hybrid assay confirmed that *Mc*HB7 can bind the ABA-related *cis*-acting elements. All of the results suggest that *Mc*HB7 may participate in the ABA signaling pathway, especially under stress conditions. The yeast two-hybrid is based on the properties of eukaryotic cell TFs such as GAL4. GAL4 consists of two domains including the DNA binding domain (BD) and activation domain (AD); both are needed for function. The TF could be constructed into the BD vector without the AD vector. If it has transaction activity, the downstream reporter gene can be activated and expressed. In our study, a non-conserved domain from 131 to 268 aa in the C-terminus acted as a potent activator, mediating the interaction between *Mc*HB7 and its interacting proteins. Metabolomics focuses on profiling and quantifying low-molecular-weight metabolites [47]. Here, 514 metabolites were identified, most of which were involved in the biosynthesis of zeatin, biotin, and proline. Interestingly, the ABA content was significantly elevated in the OE under normal and drought stress conditions compared with the WT. These results indicate that *McHB7* may participate in the growth and development of ice plants through the ABA pathway and other pathways, especially under stress conditions. Multi-omics has provided more information for us to explore the functions of proteins and metabolites as well as the molecular mechanisms underlying the plant response to abiotic stresses.

## 4. Materials and Methods

### 4.1. Plant Materials and Growth Conditions

*Mesembryanthemum crystallinum* seeds were sowed into a moist germination soil (Scotts Co., Groveland, FL, USA) in a growth chamber at a 12-h (26 °C) light/12-h dark (18 °C) cycle. Seven days later, the seedlings with four true leaves were transplanted into 946 mL foam cups and irrigated daily with 50 mL 0.5 × Hoagland’s solution [48]. The second pair of leaves from one-month-old ice plants were used for *Agrobacterium* infiltration. The infiltrated leaves were collected for further analysis.

### 4.2. Transformation of Ice Plant Leaves with pCAMBIA1300-GFP

*Agrobacterium* GV3101 with *pCAMBIA1300-GFP* was grown in 25 mL Luria Broth (LB) medium (1% bactotryptone, 0.5% yeast extract, and 0.5% NaCl) with 50 mg/L kanamycin and 25 mg/L rifampicin overnight at 28 °C in a rotary shaker at 150 rpm. The cells were collected by centrifugation at 10,000 rpm for 10 min at room temperature, and then resuspended into a 154 mM NaCl solution with 100 µM acetosyringone (As). The OD_600_ of the bacteria suspension was adjusted to 0.8 for infiltration. To test whether the protein was expressed in the ice plant leaves and whether the protein could be transferred to other leaves, the second pair of expanded leaves were injected with 500 µL of the agrobacterial suspension using a plastic needleless syringe, then the infiltrated and the non-infiltrated leaves were collected separately at 10 am for further identification (Figure 1A). Additionally, the stem was injected, and the second pair of leaves were collected (Figure 1B). To determine the transformation efficiency in ice plants, the GFP signal from day 0 (D0) to day 28 (D28) was collected at 10 am. Scotch tape was used to remove the lower epidermis of the infiltrated leaves to observe the GFP signal under a confocal laser scanning microscope (Leica DFC 7000 T, Wetzlar, Germany).

### 4.3. Western Blot and RT-qPCR to Identify Transgenic Ice Plants

To identify transgenic ice plants and estimate the transformation efficiency of *McHB7* in ice plant leaves, a recombinant vector *pCAMBIA1300-McHB7-FLAG* was constructed. Briefly, the full length of *McHB7* (804 bp) was cloned from ice plant leaves with primers *McHB7F* and *McHB7R* (Supplementary Table S1). Next, *McHB7* was used as a template, and *fMcHB7F* and *fMcHB7R* with restriction enzyme sites *Bam* HI and *Xba* I, respectively, and *fMcHB7R* with 3 × FLAG (DYKDDDDK) were used as specific primers to amplify *McHB7* and then ligated into the plant binary vector *pCAMBIA1300*. Please refer to Supplementary Table S1 for the primers used. The FLAG signal was detected by Western blot analysis, as previously described [13]. To estimate the *McHB7* expression, the infiltrated leaves with *McHB7-pCAMBIA1300-FLAG* were collected from D0 to D28 at 10 am for RNA extraction and then reverse transcription into cDNA. qPCR using the SYBR Green detection system (Bio-Rad, Hercules, CA, USA) was used to determine the relative expression levels of *McHB7*. C_t_ values in each case and a standard curve for each primer pair were obtained based on the values of the input serial dilution. The primers for qPCR are listed in Supplementary Table S1. The 2^−ΔΔCt^ method [49] and Student’s *t*-test [50] were used for data analysis.

### 4.4. Drought Stress Treatment

One-month-old ice plants were infiltrated with *Agrobacterium* harboring *pCAMBIA1300-McHB7-FLAG* (OE plant) or *Agrobacterium* without the plasmid (WT plant). Since the target protein had the highest expression level in the infiltrated leaves at day 7, these infiltrated leaves were used as materials for the drought stress experiments. Under control conditions, each plant was irrigated with 50 mL 0.5 × Hoagland solution. For drought treatment, the plants were first given adequate 0.5 × Hoagland solution, then were withheld from water after infiltration for seven days. One gram of infiltrated leaves was collected for protein extraction, as previously described [13]. For Western blot, 20 μg of each protein sample was used, as previously described [13]. For quantification of the *McHB7* expression level under drought treatment, 100 mg of infiltrated leaves was collected for RNA extraction and then transcribed into cDNA for qPCR.

### 4.5. Morphological and Physiological Measurements

The OE and WT ice plants were treated with drought for seven days, and the area of infiltrated leaves was measured by Image J 1.54d [51]. Ten leaves were collected at 10 am for each sample. To obtain the fresh weight of leaves, the infiltrated leaves were detached and weighed. The dry weight of each leaf was measured after being dried in an 80 °C oven for 24 h. The relative water content (RWC) was calculated as follows: RWC = (fresh weight − dry weight)/fresh weight × 100%. Each sample had four biological replicates. The physiological parameters included superoxide dismutase (SOD), peroxidase (POD), and malondialdehyde (MDA), which were assayed as previously described [41]. For ascorbate peroxidase (APX) measurement, 0.1 g of the leaf sample was homogenized in liquid nitrogen and transferred to a tube with 3 mL 50 mM cold PBS buffer (1 M K_2_HPO_4_ and 1 M KH_2_PO_4_, pH 7.8) with 0.4% (*w*/*v*) polyvinyl pyrrolidone, then placed on an agitator at 4 °C for 1 h. The samples were centrifuged at 5000 rpm at 4 °C for 10 min and the supernatant was the crude enzyme solution. The enzyme solution of 0.1 mL was mixed with 1.7 mL 50 mM PBS (pH 7.0) containing 0.1 mM EDTA-Na_2_ and 0.1 mL 0.5 mM ascorbic acid. Finally, 0.1 mL 0.1 mM H_2_O_2_ was added, and the absorbance was recorded quickly at a wavelength of 290 nm. The APX activity (μM/g/min) was calculated through ΔA_290_ × V/(A × v × W × Δt), where ΔA_290_ represents the value changes in 1 min at 290 nm, V is the extraction volume, and v is the reaction volume. A is an extinction coefficient of 2.8 mM^−1^cm^−1^. Δt is the reaction time. The catalase activity (CAT) was assayed according to the method of Shim [52]: 0.1 mL of the above enzyme extraction was used and the changes in OD value at a 240 nm wavelength were monitored in a reaction mixture of 50 mM PBS (pH7.0), 50 μM H_2_O_2_, and 10% H_2_SO_4_. For the chlorophyll assay, 0.3 g of leaf was collected and ground into powder with a mortar and pestle. The chlorophyll was extracted with 1 mL 80% acetone for 30 min each time for a total of three times. All extracts were combined. The total chlorophyll (mg/g) was calculated through (20.29A_645_ + 8.04A_663_) × V/W, where A_663_ and A_645_ are the absorbance at the 663 nm and 645 nm wavelengths, respectively. V is the final volume of chlorophyll extract, and W is the fresh weight of leaves.

For histochemical analyses including Evans blue, nitroblue tertazolium (NBT), and 3,3′-diaminobenzidine (DAB) staining, the infiltrated leaves were punched into 2 cm diameter circles. The samples were completely immersed into 1 mg/mL Evans blue stain, DAB solution (pH 3.8), and NBT (pH 7.8), respectively, and incubated at 37 °C overnight. Then, the samples were washed three times with distilled water and destained with 75% ethanol.

### 4.6. Liquid Chromatography-Mass Spectrometry (LC-MS/MS) and Data Analysis

To determine the protein changes in the *McHB7* transgenic ice plant leaves after the seven day stress treatment, the leaves at day 0 and day 7 were used for protein extraction, digestion, and LC-MS/MS analysis [13]. Briefly, LC-MS/MS was performed on an Easy-nLC 1200 system (Thermo Fisher Scientific Inc., Germering, DE, USA) coupled with a Q-Exactive HF Orbitrap mass spectrometer (Thermo Fisher Scientific Inc., San Jose, CA, USA). The LC method was as follows: 0–5 min, 2% B; 140 min, 35% B; 160 min, 100% B; 165 min, 100% B; 170 min, 2% B; 180 min, 2% B, run stop. The MS parameters were the same as previously described [13]. For the data analysis, Proteome Discoverer™ 2.5 (Thermo Fisher Scientific, Bremen, Germany) was used for protein identification, quantification, and functional categorization. The data were normalized by median and generalized logarithm transformation (Log2). GO enrichment analysis was conducted using WEGO 2.0 (https://wego.genomics.cn/, accessed on 15 November 2023). The volcano plots were created by GraphPad Prism 9, and the Venn diagram was generated by online software VENNY 2.1 (https://bioinfogp.cnb.csic.es/tools/venny/index.html, accessed on 15 November 2023).

### 4.7. Yeast One-Hybrid Assay and In Vivo Transactivation Assay

To test whether *Mc*HB7 can bind to *cis*-acting elements like the ABA response motifs ACGTA and TGTCG, the Early Responsive to Dehydration (ERD) motif TGTCA, and the ABA-related motif CACAT [23], each motif was repeated three times. *Hind* Ⅲ and *Sac* I restriction sites were added to the primer sequences (Supplementary Table S1) to synthesize complementary DNA strands. Then, the triple tandem of each element was inserted to the pAbAi yeast reporter vector (Figure 4A), which is usually used in one-hybrid assays for identifying and characterizing the DNA-binding proteins. These vectors were named pAbAi-ACGTA, pAbAi-TGTCA, pAbAi-TGTCG, and pAbAi-CACAT, respectively. The full-length of *McHB7* was ligated to the pGADT7 (AD) vector named AD-*Mc*HB7. The linearized plasmids pAbAi-ACGTA, pAbAi-TGTCA, pAbAi-TGTCG, and pAbAi-CACAT, which were digested by *Bstb* I at 65 °C for 15 min, were transformed into Y1H Gold yeast competent cells. AD-*Mc*HB7 was transformed into the pAbAi-ACGTA, pAbAi-TGTCA, pAbAi-TGTCG, and pAbAi-CACAT yeast competent cells, respectively, and cultured on selective medium without Ura and Leu, or without Ura and Leu but containing 250 ng/mL AbA (Takara, Beijing, China) at 30 °C for four days. pGADT7-53/pAbAi-p53 was used as the positive control, and AD-McHB7/pAbAi-p53 was used as the negative control.

To explore the transactivation domain of *Mc*HB7, the full-length coding region of *Mc*HB7 from 1 to 68 aa and the truncated sequences including the N-terminal sequence 1–38 aa, the C-terminal sequence containing conserved domain HD and HALZ 39–268 aa, the terminal sequence containing HD 1–89 aa, the C-terminal sequence containing HALZ 90–268 aa, the N-terminal sequence containing HD and HALZ 1–130 aa, and the C-terminal sequence 131–268 aa were inserted into the *pGBKT7* vector (BD), respectively. These recombinant constructs were named BD-*Mc*HB7, BD-*Mc*HB7a, BD-*Mc*HB7b, BD-*Mc*HB7c, BD-*Mc*HB7d, BD-*Mc*HB7e, and BD-*Mc*HB7f, respectively (Figure 4C). The primers for vector construction are listed in Supplementary Table S1. The empty vector pGBKT7 was used as the negative control and pGBKT7-53/pGADT7-T was used as the positive control. All of the above constructs were transformed into the Y2H yeast strain and screened on the selective dropout medium SD without Trp, and the His. *β*-Galactosidase assay was performed on filter lifts of the colonies to detect the activation of the *lacZ* reporter gene.

### 4.8. Immunoprecipitation Coupled with LC-MS/MS

The *Mc*HB7 immunoprecipitation (IP) experiment was performed according to a previous method [53] with minor modifications. Briefly, one-month-old ice plant leaves were infiltrated with *pCAMBIA1300-McHB7-FLAG* (OE) and grown for one week. One gram of the infiltrated leaves was ground into powder in liquid nitrogen, immersed in 500 μL protein extraction buffer (150 mM NaCl, 50 mM Tris-HCl, pH 8.0, 1 mM EDTA, 1% Triton X-100 and 200 μL 100 × protease cocktail) and vortexed at 4 °C for 1 h. WT was used as the control. The crude protein extraction was centrifuged at 10,000 rpm for 10 min. The collected suspension was incubated with 10 μL anti-FLAG M2 resin (Sigma, St. Louis, MO, USA) at 4 °C for 2 h on a rotary shaker. The mixture was centrifuged at 10,000 rpm for 30 s and washed with TBS buffer (150 mM NaCl and 50 mM Tris-HCl (pH8.0)) three times, each time for 5 min. The bound proteins were eluted by adding 100 μL elution buffer (protein extraction buffer with 200 μg/mL 3 × FLAG peptide (Sigma, St. Louis, MO, USA)) and shaken at 4 °C for 30 min. After centrifugation at 10,000 rpm for 1 min, the supernatant was collected. To test the efficiency and quality of the IP, 20 μg of protein was used to run the SDS-PAGE gel and Western blot, as previously described [12,13]. The protein samples were then digested with 1:50 (*w*/*w*) trypsin at 37 °C overnight. The digested solution was collected in a new tube and 10 μL 20% (*v*/*v*) formic acid was added. The tubes were then placed in a Thermo Scientific Savant SpeedVac for lyophilization for 2 h. Each sample was resuspended with 20 μL 0.1% (*v*/*v*) formic acid, and then cleaned up using ZipTip^®^ C18 pipette tips (Sigma Millipore Ltd., St. Louis, MO, USA). First, the ZipTip was activated by 100% acetonitrile once, and 50% acetonitrile for second time. Then, the ZipTip was equilibrated with 0.1% formic acid 8–10 times. Next, the peptides were aspired through the ZipTip for 8–10 times and washed with 0.1% formic acid for another 8–10 times. Finally, the peptides were eluted by 30 μL 80% acetonitrile and 0.1% formic acid and dried with a SpeedVac for 30 min. Each sample was resuspended in 15 μL 0.1% formic acid for LC-MS/MS, and each had four replicates.

### 4.9. Metabolite Extraction and Metabolomic Analysis

Four biological replicates of the leaves were freeze-dried by a vacuum lyophilizer for 48 h, and 10 mg dry weight of each sample was put in a 2 mL sterilized tube. Internal standards of 100 μM lidocaine and 100 μM (1S)-(+)-10-camphorsulfonic acid were added to the leaf samples as the positive and negative internal references, respectively. First, 0.5 mL solution Ⅰ (acetonitrile/isopropanol/water 3:3:2 (by volume)) was added to each sample and vortexed at 200 rpm, 4 °C for 5 min, then sonicated on ice for 15 min and centrifuged at the speed of 13,000 rpm at 4 °C for 20 min. The supernatant was transferred to a new tube. Second, 0.5 mL solution Ⅱ (acetonitrile/water 1:1 (by volume)) was added and the previous extraction procedure was repeated. Third, solution Ⅲ (80% methanol) was added for metabolite extraction following the previous procedure. Finally, the supernatant was combined and lyophilized to dryness. To resolubilize the metabolites, 100 μL 30% methanol and 0.1% formic acid (by volume) was added to the dried metabolite samples and sonicated for 20 min, followed by centrifugation at 13,000 rpm for 20 min. The supernatant was used for untargeted metabolomics on a high-resolution Orbitrap Fusion Tribrid mass spectrometer coupled with a Vanquish™ UHPLC system. Both positive and negative modes were employed to obtain the MS1 accurate mass and MS2 product fragments. The flow rate was set to 0.45 mL/min. The LC gradient was as follows: 0 min, 0% of solvent B (i.e., 100% of solvent A); 21 min, 40% of solvent B; 23min, 95% of solvent B; 24 min, 95% of solvent B; 25 min, 0% of solvent B; 30 min, stop run. For data analysis, Compound Discoverer™ 3.0 software from Thermo Fisher Scientific was used to identify and quantify the metabolites.

### 4.10. Statistical Analysis

The data were collated using WPS office 2019 software [54] and SPSS 26 software [55] for one-way ANOVA statistical analysis and the least significant difference (LSD) method for multiple comparisons, with significant differences defined at *p* < 0.05.

## 5. Conclusions

In this study, the long-term overexpression of the *McHB7* gene in ice plant leaves through the *Agrobacterium*-mediate transformation was achieved. This system allows for gene functional testing in the same native plant, rather than the commonly used heterologous plants such as tobacco and Arabidopsis. Under drought stress, the morphological phenotype and physiological indices indicate that the OE plants were highly responsive to stress compared with the WT, resulting in improved stress tolerance. *Mc*HB7 can bind to ERD and ABA-related *cis*-acting elements and has self-activation with the potent activity domain located from 131 aa to 268 aa. The *Mc*HB7 associated proteins are involved in stress response, metabolite turnover, and hormone (e.g., ABA) biosynthesis. Taken together, these results provide important information about the multi-functions of *McHB7*. Several lines of evidence establish that one of the *McHB7* functions is to regulate the ABA pathway. In addition, *McHB7* affects cellular redox homeostasis and carbohydrate and energy metabolism. Therefore, the *McHB7* TF may be a master regulator in ice plant stress response and tolerance as well as in its transition from C_3_ to CAM to enhance stress resilience.

## Data Availability

The proteomics raw data have been deposited at the ProteomeXchange via PRIDE with the identifier PXD033521. The metabolomics raw data have been deposited at the MetaboLights data repository with the identifier MTBLS4744.

## Supplementary Materials

The following supporting information can be downloaded at: https://www.mdpi.com/article/10.3390/ijms25084569/s1.
