# Supplementary material for: Transcription Factor McHB7 Improves Ice Plant Drought Tolerance through ABA Signaling Pathway
Source: Int J Mol Sci. 2024 Apr 22;25(8):4569. doi: 10.3390/ijms25084569 (PMC11050535; doi:10.3390/ijms25084569)
Supplement: Supplementary file 1 [file ijms-25-04569-s001.zip › ijms-2961609-supplementary.pdf]

## Supplementary figures and tables:

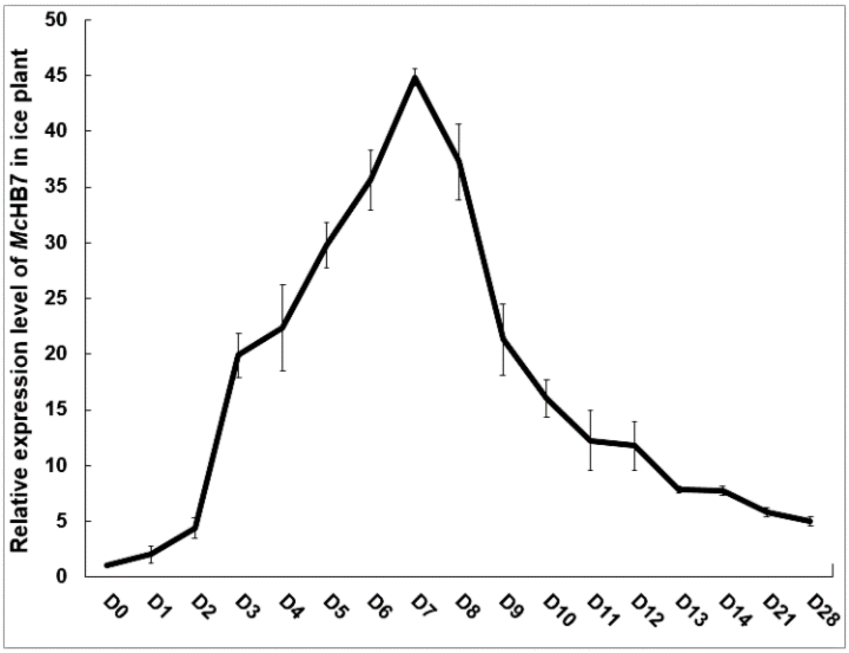


**Figure S1.** Relative expression level of *McHB7* gene in infiltrated ice plant leaves; D0-D28, The infiltrated leaves from day 0 to day 28.

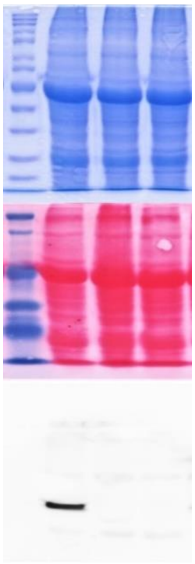

**Figure S2.** Original gel and Western images of the results shown in different main Figures.

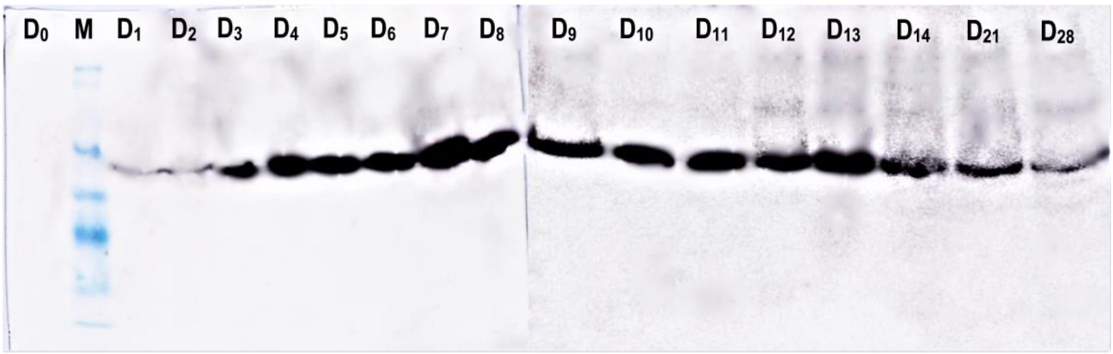


**Figure 1C**. Western blot analysis of extracted proteins from the ice plant leaves 1–3.

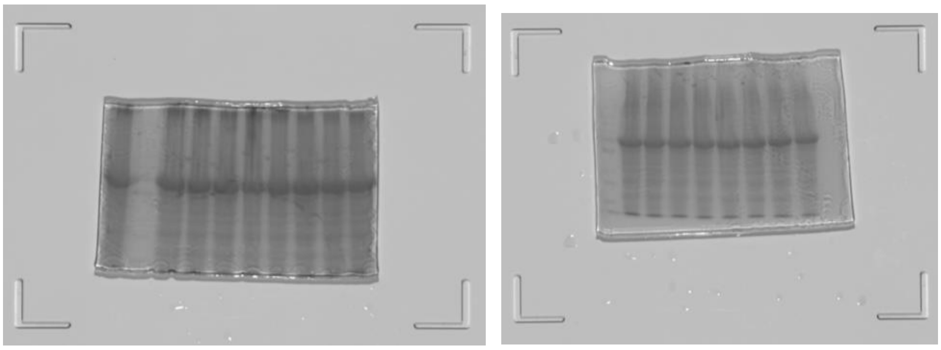

**Figure 1E**. Western blot of transgenic ice plant leaves from day 0 (D0) to day 28 (D28).

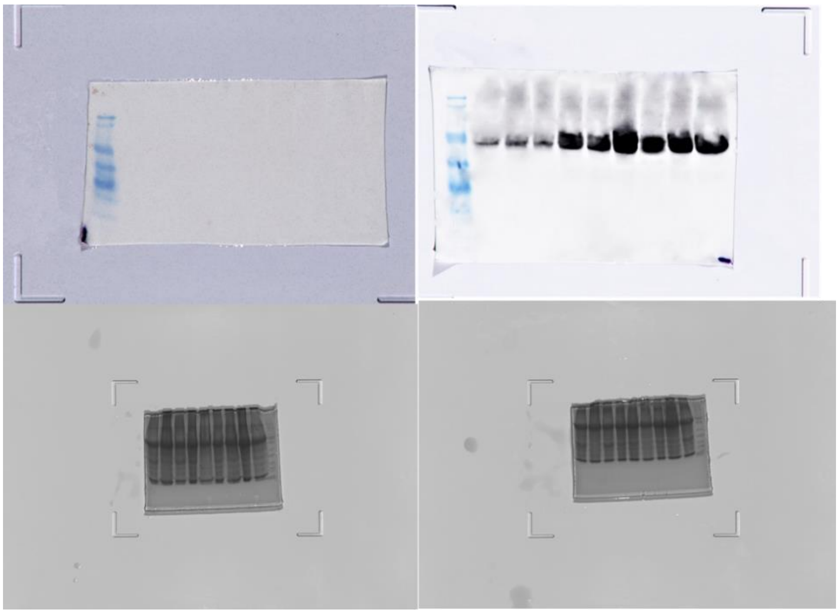

**Figure 2D**. *Mc*HB7 protein levels in the OE under control and drought stress compared with WT.

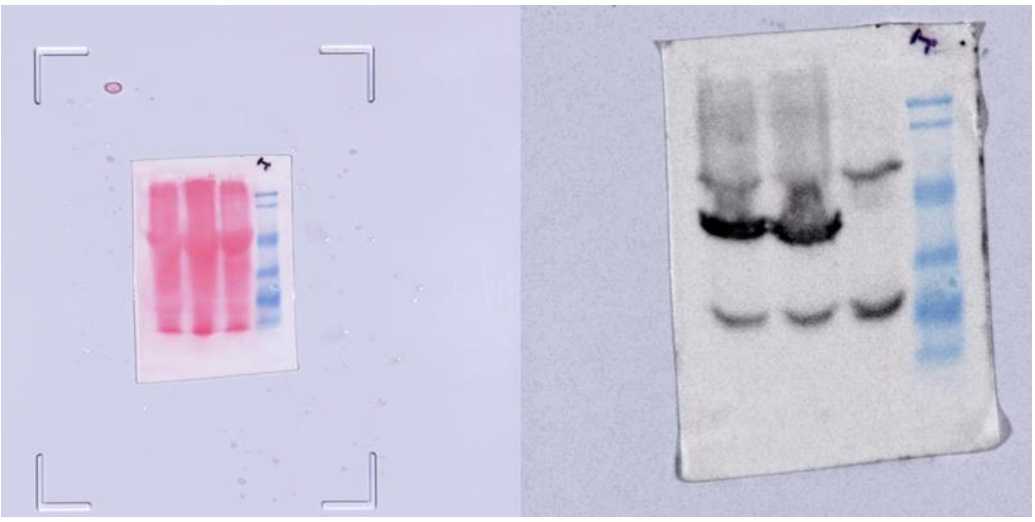

**Figure 7A**. IP result with the *Mc*HB7 bait.

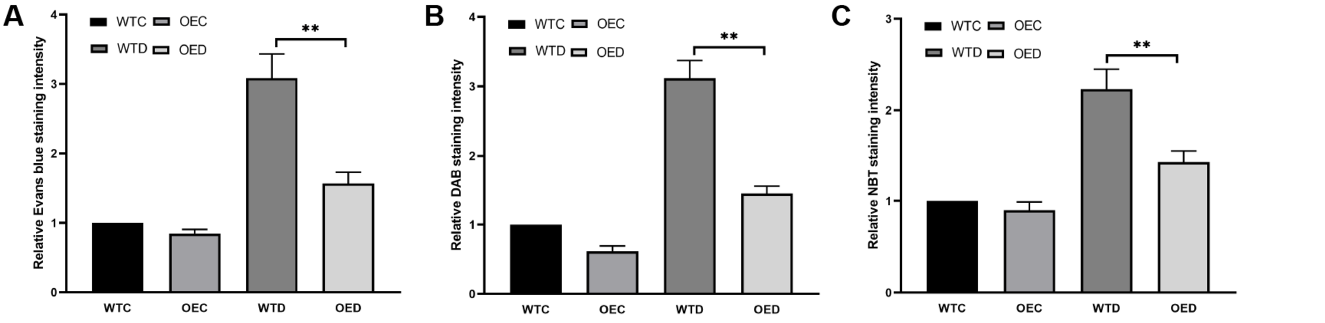


**Figure S3.** Data analysis of Evans blue, DAB and NBT staining corresponding to Figure 3H; (**A**) Relative Evans blue staining intensity; (**B**) Relative DAB staining intensity; (**C**) Relative NBT staining intensity.

**Supplementary Table S1.** List of primers, their sequences and purpose for this study.

| Primer Name | Primer Sequence (5′-3′) | Purpose |
|---|---|---|
| *fMcHB7F* | CGCGGATCCATGATGTATGAAGAAGGAGAA | For the construction of *McHB7-pCAMBIA1300-FLAG* |
| *fMcHB7R* | CGGTCTAGATCAGGCGCCTTTGTCATCGTCATCCTTGTAG-TCTCCGCCTTTATCGTCATCGTCTTTATAATCTCCGCCTTTGTCATCGTCATCCTTGTAGTCTCCGCCCGACCAAAATTCCCACCATTG | |
| *McPIP1;2F* | GATGCCAAGAGGAGTGCTAG | Internal primers used for RT-qPCR |
| *McPIP1;2R* | GTTCCAGTGATTGGGATGGTT | |
| *rtMcHB7F* | CGAGACGAACAGCAATAATAGT | Primers for *McHB7*quantification by RT-qPCR |
| *rtMcHB7R* | CTTCACACACATCGCATTTCT | |
| *AD-McHB7F* | GGAATTCATGATGTATGAAGAAGGAGAA | Primers used for the construction of *AD-McHB7* for yeast one-hybrid assay |
| *AD-McHB7R* | CGAGCTCTCACGACCAAAATTCCCACCA | |
| *p1F* | AGCTTACGTAACGTAACGTAGAGCT | Primers used for the construction of *pAbAi-ACGTA* |
| *p1R* | TTACGTTACGTTACGTG | |
| *P2F* | AGCTT TGTCATGTCATGTCAGAGCT | Primers used for the construction of *pAbAi-TGTCA* |
| *P2R* | TTGACATGACATGACAG | |
| *P3F* | AGCTTTGTCGTGTCGTGTCGGAGCT | Primers used for the construction of *pAbAi-TGTCG* |
| *P3R* | TCGACACGACACGACAG | |
| *P4F* | AGCTTCACATCACATCACATGAGCT | Primers used for the construction of *pAbAi-CACAT* |
| *P4R* | TATGTGATGTGATGTGG | |
| *BD-McHB7F* | CCATGGCCATGATGTATGAAGAAGGAGAA | Primers used for the construction of BD-*Mc*HB7 for transactivation assay |
| *BD-McHB7R* | GTCGACTCACGACCAAAATTCCCACCA | |
| *BD-McHB7aF* | CCATGGCCATGATGTATGAAGAAGGAGAA | Primers used for the construction of *BD-McHB7a* |
| *BD-McHB7aR* | GTCGACTCTCTTGTTCATCATCTTCTT | |
| *BD-McHB7bF* | CCATGGCCCGATTCAGCGACGATCAAGTG | Primers used for the construction of *BD-McHB7b* |
| *BD-McHB7bR* | GTCGACTCACGACCAAAATTCCCACCA | |
| *BD-McHB7cF* | CCATGGCCATGATGTATGAAGAAGGAGAA | Primers used for the construction of *BD-McHB7c* |
| *BD-McHB7cR* | GTCGACCTTGTAACGAGCCCTCTTGTT | |
| *BD-McHB7dF* | CCATGGCCAAGCAGCTAGAGCACGAGTAT | Primers used for the construction of *BD-McHB7d* |
| *BD-McHB7dR* | GTCGACTCACGACCAAAATTCCCACCA | |
| *BD-McHB7eF* | CCATGGCCATGATGTATGAAGAAGGAGAA | Primers used for the construction of *BD-McHB7e* |
| *BD-McHB7eR* | GTCGACATCTTTCAACTTCTGTATCTG | |
| *BD-McHB7fF* | CCATGGCCATAGCGGCGAGGAAGGGAGAA | Primers used for the construction of *BD-McHB7f* |
| *BD-McHB7fR* | GTCGACTCACGACCAAAATTCCCACCA | |
